# Balanced time perspective, time management disposition, and resilience: a moderated mediation model of academic performance

**DOI:** 10.3389/fpsyg.2025.1484152

**Published:** 2025-03-11

**Authors:** Yan Li

**Affiliations:** College of Educational Sciences, Luoyang Normal University, Luoyang, China

**Keywords:** balanced time perspective, academic performance, time management disposition, resilience, psychological traits

## Abstract

**Objective:**

Time perspective and time management disposition are critical factors influencing academic achievement. Although balanced time perspective (BTP) has been associated with adaptive functioning across various life domains, its relationship with academic performance remains underexplored. This study proposes a moderated mediation model to examine the link between BTP and academic performance.

**Methods:**

The study included 1,076 high school students (448 boys and 628 girls), aged 15 to 19 years. Participants completed self-report questionnaires assessing BTP, time management disposition, and resilience in their classrooms. Academic performance was measured using standardized test scores routinely administered by the school. The valid data were analyzed using the PROCESS macro for SPSS.

**Results:**

BTP positively predicted academic performance, with time management disposition mediating this relationship. The indirect effect was further moderated by resilience, demonstrating a stronger effect among students with higher resilience levels.

**Conclusion:**

These findings suggest that BTP may influence children’s academic performance and offer novel strategies for promoting academic achievement in high school settings. In addition, the findings highlight the importance of fostering psychological traits like resilience to enhance academic performance. Future studies could explore educational interventions aimed at developing BTP and resilience, thereby enhancing students’ psychological skills.

## Introduction

Time awareness is critical for a variety of kinds of performance (Varlamova, 2008), including academic performance (Nuttin, 1985; Rudzińska-Wojciechowska et al., 2021). Two time-related factors have been studied in research on academic success. First, time management predicts academic performance (Britton and Tesser, 1991; Deng, 2005; Ghamari et al., 2013; Razali et al., 2018; Zhang et al., 2001), as does time management as a disposition (Li, 2017; Ruan and Deng, 2004; Zhang et al., 2001). Second, time perspective is the “nonconscious process whereby the continual flows of personal and social experiences are assigned to temporal categories, or time frames, that help to give order, coherence, and meaning to those events” (Zimbardo and Boyd, 1999, p. 1271), it has been shown to predict academic performance (Rudzińska-Wojciechowska et al., 2021; Barnett et al., 2020).

Balanced time perspective (BTP) is a significant trend in the field of time perspective (Webster and Ma, 2013), and it is the key to a fulfilling existence (Boniwell and Zimbardo, 2003). As an optimal time orientation, BTP is the combination of different time perspectives (the past, present, and future) based on situational demands, a person’s needs, and values (Zimbardo and Boyd, 1999), and it may also be related to academic performance, though the evidence for this assertion is primarily indirect. BTP has been demonstrated to be adaptive in many parts of life (Stolarski et al., 2020), and it is thought to aid in problem solving and coping with life’s challenges (Witowska and Zajenkowski, 2019; Zajenkowski et al., 2016a). It has been linked to improved work-life balance and well-being (Boniwell, 2005), positive emotional states (Syrtsova and Mitina, 2008; Boniwell et al., 2010), self-esteem, clear life objectives, and a feeling of direction (Syrtsova and Mitina, 2008).

In certain situations, each time perspective may be applicable (Zimbardo and Boyd, 1999), but overuse or underuse of any one time perspective may result in cognitive biases (Zimbardo and Boyd, 1999; Boniwell and Zimbardo, 2004), affecting goal-directed behaviors and resulting in negative outcomes (Barnett et al., 2020). Learning to overcome the imbalance and develop a balanced temporal perspective should be a requirement for all of us (Boniwell and Zimbardo, 2003; Boniwell and Zimbardo, 2004).

For high school students, learning tasks are heavy and learning time is limited, the majority of studies investigating time perspective have focused on the future perspective (Webster, 2011), lack in-depth discussion on the internal mechanism of the relationship between BTP and academic performance. This study delves into the relationship between the variables and the underlying mechanisms of influence.

### Balanced time perspective and academic performance

Despite the fact that there is more research on BTP, most of it focuses on its connection to mental health or well-being, there is less research on its connection to academic performance.

Balanced time perspective has been found to be associated with many factors that are important for academic performance, including executive control (Zajenkowski et al., 2016b), the use of deeper and repetitive learning strategies to process learning material (Horstmanshof and Zimitat, 2007), and higher engagement when performing difficult cognitive tasks. Individuals with BTP have positive attitudes about life and themselves (Sobol and Jankowski, 2016), greater optimism, and a stronger sense of self-efficacy (Boniwell et al., 2010). They have life goals (Syrtsova and Mitina, 2008), make better use of time (Boniwell and Osin, 2015), and manage it more effectively (Harber et al., 2003). BTP is associated to self-regulated learning (de Bilde et al., 2011). Individuals with BTP enjoy the process of learning and are more creative (Boniwell and Zimbardo, 2004).

Balanced time perspective has also been linked to increased fluid intelligence (Zajenkowski et al., 2016a; Zajenkowski et al., 2016b), which is one of the most significant variables in learning (Jaeggi et al., 2008; Schweizer and Koch, 2001). The theory of fluid and crystallized intelligence (Cattell, 1963) states that individual differences in knowledge and skill acquisition are largely influenced by the amount of fluid intelligence invested in learning (Kvist and Gustafsson, 2008). Previous studies, such as that of Diotaiuti et al. (2021), have demonstrated the role of psychological traits, including emotional balance, in mediating the relationship between procrastination and academic performance. This study expands this perspective by integrating resilience as a moderating variable.

Thus, there is a need for more research to determine if BTP affects academic performance more generally. If there is an association, it would be useful to identify the mechanism of the effect. An understanding of these processes could inform efforts to promote students’ academic success.

*Hypothesis 1*: BTP will predict academic performance, the higher the level of balanced time perspective, the better the academic performance.

### The mediating role of time management disposition

Time management has been shown to predict academic performance in several studies (Britton and Tesser, 1991; Deng, 2005; Ghamari et al., 2013; Razali et al., 2018; Zhang et al., 2001). It has also been demonstrated that time management disposition (TMD), a similar factor, is linked to academic success. TMD is considered an individual difference in how people use their time (Huang and Zhang, 2001; Zhang et al., 2001). Time management disposition are motivational personality traits that motivate people to act toward certain goals (Huang and Zhang, 2001), exploring time management disposition can help improve time management skills.

#### Time management disposition and academic success

Research has shown that TMD is correlated with academic success (Ji, 2018; Du and Lyu, 2017; Nasrullah and Khan, 2015; Zhang et al., 2001), and students with high and low academic performance can be distinguished based on TMD (Du and Lyu, 2017; Ruan, 2004). In addition, students with high TMD value their time, act toward specific goals, believe that their efforts will be repaid in the long run, and allocate study time rationally (Ruan and Deng, 2004). They also exhibit greater confidence in their capacity to manage their time (Zhang et al., 2018; Kelly, 2003).

It can be seen that the effect of BTP on academic performance can be mediated by time management disposition. Students with strong balanced time perspective tend to manage their time better, make reasonable study plans, and organize their study and rest time effectively. This good time management disposition will further influence students’ learning attitudes and behaviors and improve learning efficiency and achievement.

#### Mediation process

The proposed mediation process is one in which (1) BTP is directly associated with higher academic performance, and (2) BTP is associated with higher TMD, which in turn predicts higher academic performance. The above-cited studies provide evidence to support each of these pathways. With regard to the direct association, there are numerous conceptual reasons to expect that BTP will be associated with academic performance, as well as evidence that students with BTP work more productively when studying (Boniwell and Zimbardo, 2004). Regarding the indirect association, there is evidence that BTP predicts TMD (Boniwell and Osin, 2015; Harber et al., 2003), and that TMD predicts academic success (Du and Lyu, 2017; Ji, 2018; Nasrullah and Khan, 2015; Ruan, 2004; Zhang et al., 2001).

*Hypothesis 2*: Time management disposition will mediate the association between BTP and academic performance.

### The moderating role of resilience

The definition of resilience is one’s “ability to adapt or rebound quickly from change, illness, or bad fortune” (Peccoralo et al., 2020, p. 190). Resilience is a multidimensional construct consisting of protective factors that individuals use to cope with difficult situations (DeSimone et al., 2016; Martin et al., 2015; Pangallo et al., 2015). The four components are good emotions, reason and purpose, connections with others, wellness flexibility (Peccoralo et al., 2020).

Resilience is the ability to recover from stress or adversity (Tugade, 2011). It is positively correlated with the experience of positive emotions (Folkman, 2008) and the use of adaptive coping strategies (Tugade et al., 2004), both of which have been shown to assist individuals in the process of stress recovery (Tugade and Fredrickson, 2004; Tugade et al., 2004), thereby reducing stress and anxiety (Bensimon, 2012).

Resilience, as an adaptive capacity to cope with adversity, may play a critical role in mediating the effect of BTP on academic performance, as suggested by recent studies in positive psychology (Diotaiuti et al., 2021). For high school students, life is full of challenges and resilience has a significant impact on academic success (Yeager and Dweck, 2012). The regulation of emotion and behavior, and the motivation for learning, etc. can threaten an individual’s adaptive systems (Masten, 2014). Individuals with low balance time perspective produce imbalanced time patterns (Stolarski et al., 2016). Cognitive theories of stress and coping suggest that individuals with high resilience also experience positive emotions in adversity and adopt adaptive coping strategies (Folkman, 2008; Folkman and Moskowitz, 2000). In the face of unfavorable circumstances, compared to low resilience, high resilience allows the person to use a repertoire of problem-solving skills (Tusaie and Dyer, 2004), and perceive less psychological distress (Almeida, 2005; Friborg et al., 2006). Resilience has the capacity to mitigate the adverse effects of risk factors, improve environmental adaption, and enable the efficient management of thoughts and emotions (Masten, 2014).

This evidence suggests that individuals with high resilience are able to adopt adaptive coping strategies and make reasonable time allocations. They can also self-regulate and implement time plans to achieve academic goals. This suggests that resilience may play a moderating role in the association between BTP and academic performance.

*Hypothesis 3*: The mediating role of time management disposition will be moderated by resilience. Specifically, the mediation effect will be stronger when there is high resilience.

### Research on adolescents

Adolescents are a relevant group in which to test our moderated mediation model. Regarding the association between BTP and TMD, adolescents need a new perspective on time and new skills for time management to succeed under a heavy study load (Zhao et al., 2009). The research on adults has shown that goal conflicts can make time seem scarcer (Etkin et al., 2015). The relationship between TMD and academic performance has received less attention in studies focusing on teenagers, but Ruan and Deng (2004) discovered that students with high time management disposition outperform those with low school achievement in a sample of middle school students. In addition, during adolescence resilience is associated with skills that may promote academic success, including general self-efficacy (Hamill, 2003; Lee et al., 2013), long-term goals, greater involvement in extracurricular activities, and higher school engagement (McMillan and Reed, 1993; Peck et al., 2008).

### The present study

**Figure 1:**
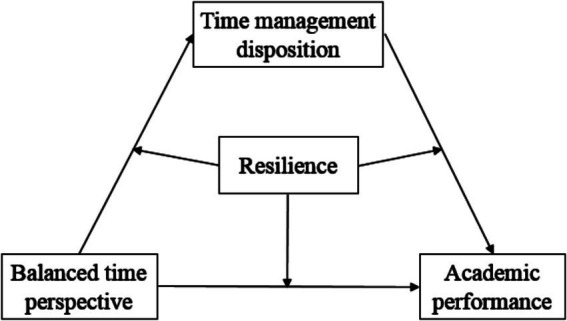
A moderated mediation model of balanced time perspective, time management disposition, resilience, and academic performance.

Based on the extant research on the predictors of academic success, we constructed and tested a statistical moderated mediation model (see Figure 1) in a large sample of Chinese high school students. The results will contribute to an understanding of the relationships among high school students’ reports of BTP, time management disposition, resilience, and academic performance.

This study examined the mechanism of the effect of BTP on the academic performance of high school students. The results highlight the benefits of helping adolescents to establish a BTP and improve time management as ways to increase academic performance.

## Methods

### Participants

Participants were 1,251 10th-12th grade students recruited from three high schools in Henan Province, China. Twenty-three participants were excluded from the sample because they showed an obvious response pattern, so the final sample consisted of 1,076 adolescents, 58.4% girls (*n =* 628), 41.6% boys (*n =* 448). The proportion of students in each grade (Grades 10–12) was 31.6% (*n =* 340), 53.8% (*n =* 579), and 14.6% (*n =* 157) respectively. The proportion of only children was 23.0% (*n =* 248), and the proportion of children with at least one sibling was 77.0% (*n =* 828). The students ranged in age from 15 to 19 years (*M* = 16.18, SD = 0.901).

As the sample is limited to three high schools in China, the findings may not be generalizable to other cultural or educational contexts. Future studies could consider more diverse samples to broaden the scope of conclusions.

### Procedure

We cooperated with the student counselors at the three schools to carry out the study, which was approved by the research ethics board of the corresponding author’s university. Written informed consent was obtained from parents and the students provided assent. Before data collection, the students were informed about the anonymity of the data and the voluntary nature of participation. Under the supervision and guidance of trained research assistants in an organized classroom setting, students filled out the self-report questionnaires regarding BTP, time management disposition, and resilience. Academic performance requires students to report grades in language, mathematics, and English at the end of the summer semester. The questionnaires took about 15 min to complete. No incentive or reward was offered.

### Measures

#### The BTP Inventory

The Balanced Time Perspective Inventory (BTPI; Li and Lyu, 2021) is a Chinese language self-report measure of students’ ability to focus on the past, present, or future depending on the situation. It comprises 28 items rated on a five-point Likert scale (1 = very non-conforming, 5 = very conforming). There are seven subscales, each with four items: past positive, past negative, present engaged, present hedonic, future positive, future negative, and excessive future orientation [Example items are “Memories of the past add joy to my life” (past positive) and “Thinking about my future makes me unhappy” (future negative)]. Each dimension score was calculated as the mean of all items, and then the dimension scores were normalized as *Z* scores with *M* = 0 and *SD* = 1. BTP was calculated as the difference between dimensions that represented positive and negative time perspectives: BTP = (Z past positive + Z present engaged + Z future positive + Z present hedonic) – (Z past negative + Z future negative + Z excessive future). Larger values of BTP represent a higher tendency, i.e., more balanced, for individuals to show adaptive time perspective in response to different scenarios. The Cronbach’s alpha coefficient for this scale in the study was 0.807.

#### The Adolescence Time Management Disposition Scale

The Adolescence Time Management Disposition Scale (Huang and Zhang, 2001) is a Chinese language self-report measure to assess adolescent time management disposition. It comprises 44 items rated on a five-point Likert scale (1 = not at all, 5 = completely) and divided into three subscales: time value (e.g., “Whatever I do, the first thing I consider is the time factor”), time monitoring (e.g., “I assign myself a learning goal every day”) and time efficacy (e.g., “I believe my scheduling is usually reasonable”). The higher the total score, the better the time management disposition. The Cronbach’s alpha coefficient of the scale in this study was 0.942.

#### The Adolescent Resilience Scale

The Adolescent Resilience Scale developed by Hu and Gan (2008) is a Chinese language self-report measure to assess adolescents’ resilience. It comprises 27 items rated on a five-point Likert scale (1 = not at all, 5 = completely) and divided into two factors: personal strength (e.g., “I find it difficult to control my unpleasant emotions”) and support (e.g., “When I have difficulties, I will take the initiative to confide in others”). The Cronbach’s alpha coefficient of the scale in this study was 0.738.

### Academic performance

Students’ test scores in language, mathematics, and English at the end of the summer semester were collected as a measure of their academic performance. Scores in each of the three subjects were standardized by grade level, and the average of the three standardized scores was used as the final indicator of academic achievement (*M* = 0, SD = 1).

Although the scales used are validated, integrating objective data, such as teacher evaluations or direct observations, could strengthen the robustness of the findings.

#### Control variables

Control variable is gender (0 = male; 1 = female).

### Data analysis

First, descriptive statistics and correlations were generated in SPSS 20.0. The PROCESS macro in SPSS (Hayes, 2013) was then used to test the mediation effect of time management disposition in the association between BTP and academic performance, and the moderating effect of resilience on the mediation effect. The choice of 5,000 bootstrap samples was made to ensure stable and reliable estimates of mediation and moderation pathways, and confidence intervals for the mediating effects were estimated using a bias-corrected non-parametric percentile bootstrap method. If the interval does not contain 0, the mediating effect is significant; if the interval contains 0, the mediating effect is not significant (Hayes, 2013). Variables other than demographic variables were standardized prior to tests of the moderated mediation model.

## Results

### Test of common method bias

To test whether the multi-item measures represented different constructs, we compared the validity of single-factor measurement models using a confirmatory factor analysis (CFA). From the fitting results, RMSEA = 0.212 > 0.08, CFI = 0.731 < 0.90, TLI = 0.641 < 0.90, and SRMR = 0.134 > 0.08, the model is poorly fitted, suggesting that there is no serious common method bias.

Except for the measure of academic performance, all data were collected through self-report questionnaires administered at the same time point. To reduce the effect of common method bias, we emphasized the confidentiality of the data when we gave instructions to the students. To conduct Harman’s single-factor test (Podsakoff et al., 2003), we conducted a factor analysis of all items from all self-report measures. The results showed that there were 18 factors with eigenvalues greater than 1, and the variance explained by the first factor was 23.66%. This value was less than the critical value of 40% (Tang and Wen, 2020), indicating that there was no serious common method bias in this study.

### Correlation analysis

There were positive, significant inter-correlations among BTP, academic performance, time management disposition, and resilience (see Table 1).

**Table 1:** Descriptive statistics and inter-correlations.

Variable	M	SD	1	2	3	4	5
1 Gender	0.58	0.49	–				
2 Balanced time perspective	0.08	0.98	0.02	–			
3 Academic performance	0.07	2.19	0.05	0.10^**^	–		
4 Time management disposition	3.37	0.52	−0.09^**^	0.45^**^	0.13^**^	–	
5 Resilience	3.44	0.34	0.03	0.15^**^	0.10^**^	0.46^**^	–

*N = 1,076. Academic performance is the standardization of grades.*

**p < 0.01, *p < 0.05.

### Testing the mediating role of TMD

The SPSS macro PROCESS developed by Hayes (2013) was used to test the hypotheses. First, Model 4 was used to test the mediating role of time management disposition in the association between BTP and academic performance. The results are shown in Table 2. BTP significantly and positively predicted academic performance (*β* = 0.10, *p* < 0.01) and time management disposition (*β* = 0.45, *p* < 0.001). When both BTP and time management disposition were included as predictors of academic performance, BTP was no longer a significant predictor (*β* = 0.03, *p* > 0.05) but time management disposition remained a significant positive predictor (*β* = 0.13, *p* < 0.001). The mediation effect was 0.07 with a 95% bootstrap confidence interval of [0.025, 0.089]. Thus, Hypothesis H1, H2 was confirmed.

**Table 2:** Analysis of mediation effects.

Predictor	Model 1: Academic performance		Model 2: Time management disposition		Model 3: Academic performance	
	*β*	*t*	*β*	*t*	*β*	*t*
Gender	0.09	1.51	−0.22	−3.91^***^	0.12	1.95
BTP	0.10	2.83^**^	0.45	16.73^***^	0.03	0.86
Time management disposition					0.13	3.68^***^
*R^2^*	0.01		0.21		0.02	
*F*	5.24^**^		146.23^***^		8.05^***^	

*N = 1,076, BTP, balanced time perspective.*

***p < 0.001, **p < 0.01, *p < 0.05.

### Test of the moderating effect of resilience on the mediating effect of TMD

Moderated mediation was tested using Model 59 of the PROCESS macro in SPSS. The results (see Table 3) showed that the predictive effect of BTP on time management disposition in Model 1 was significant (*β* = 0.39, *p* < 0.001), the interaction of BTP and resilience was significant in predicting time management disposition (*β* = 0.07, *p* < 0.01).

**Table 3:** Analysis of moderated mediation.

Predictor	Model 1 (time management disposition)		Model 2 (academic performance)	
	*β*	*t*	*β*	*t*
Gender	−0.23	−4.69^***^	0.11	1.75
BTP	0.39	16.13^***^	0.03	0.95
Time management disposition			0.11	2.76^**^
Resilience	0.43	16.63^***^	0.03	0.64
BTP * Resilience	0.07	2.62^**^	−0.06	−1.74
Time management disposition * Resilience			0.02	0.72
*R^2^*	0.38		0.03	
*F*	164.21^***^		4.88^***^	

*N = 1,076. BTP, balanced time perspective.*

***p < 0.001, **p < 0.01, *p < 0.05.

In Model 2, the predictive effect of TMD on academic performance was significant (*β* = 0.11, *p* < 0.01), there is no significant interaction between BTP and resilience on academic performance (*β* = −0.06, *p* > 0.05), nor between time management disposition and resilience (*β* = 0.02, *p* > 0.05). It follows that the moderating effect of resilience occurs only in the first half of the mediating effect, i.e., the effect of BTP on TMD is moderated by resilience. These results suggest that resilience amplifies the positive effect of BTP on TMD. This indicates that educational interventions aimed at developing resilience could significantly enhance students’ time management skills. Therefore, Hypothesis H3 was supported.

**Figure 2:**
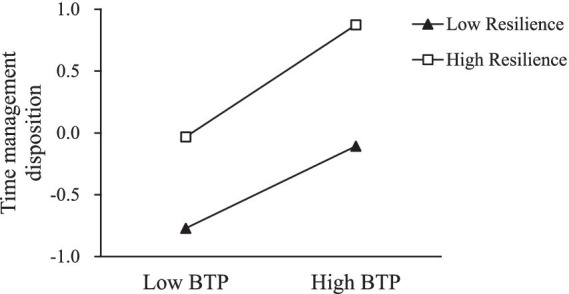
The moderating role of resilience in the relationship between time perspective and time management disposition. BTP, balanced time perspective.

To interpret the interaction effects, the students were divided based on having high (1 *SD* above the *M*) or low (1 *SD* below the *M*) resilience scores. Simple slope test was conducted to plot the moderating effects. The positive predictive effect of BTP on time management disposition was significant both at the low level of resilience (*β*_simple_ = 0.33, *t* = 9.969, *p* < 0.001) and at the high level of resilience (*β*_simple_ = 0.45, *t* = 13.366, *p* < 0.001). Besides, the slope of the association between BTP and time management disposition was steeper at the high level of resilience. Therefore, resilience can strengthen the positive predictive effect of BTP on time management disposition (see Figure 2).

As shown in Figure 2, students with high resilience benefit more from BTP in terms of time management disposition, underscoring the role of resilience in educational contexts.

## Discussion

### The mediating effect of TMD in the association between BTP and academic performance

The present study revealed that BTP did not exert a direct effect on academic performance but was indirectly associated with academic performance through the mediating role of TMD. Specifically, higher levels of BTP were associated with enhanced TMD, which in turn predicted improved academic performance. This finding aligns with prior research demonstrating the interplay between temporal cognition and academic achievement (Boniwell et al., 2014).

Empirical evidence consistently indicates that high school students often exhibit limited time-management skills (Kisa and Ersoy, 2005; Das and Bera, 2021). To address this, fostering effective time-management strategies, cultivating a positive reflection on past experiences, and proactively adjusting learning behaviors can significantly enhance academic efficiency. Importantly, proficient time management not only facilitates focused learning but also reduces academic-related stress, thereby promoting overall well-being among high school students (Das and Bera, 2021).

Effective time management has been consistently linked to enhanced quality of life (Cyril, 2015). As a critical component of self-management (Subramanian, 2016; Cyril, 2015), time management enables individuals to navigate conflicting temporal demands and mitigate stress (Subramanian, 2016). However, temporal awareness often operates at an unconscious level, with most individuals seldom engaging in metacognitive reflection on their perceptions of the past, present, and future (Zimbardo and Boyd, 2008). Developing a BTP requires cultivating temporal flexibility and the ability to adapt to situational demands. A cognitive evaluation of time allocation can facilitate the alignment of short-term actions with long-term objectives (Beracci et al., 2022), positioning BTP as a cornerstone of emotional well-being (Boniwell and Zimbardo, 2004). Empirical evidence further demonstrates that individuals with high BTP exhibit greater positive affect (Stolarski et al., 2014), reduced stress and anxiety (Papastamatelou et al., 2015), and more adaptive personality traits.

Time perspective encompasses cognitive, emotional, and social factors that influence an individual’s attention, perception, decision-making, and behavior (Boniwell and Zimbardo, 2004). Research indicates that individuals with a BTP demonstrate more accurate time perception and improved temporal awareness (Witowska et al., 2020). Effective TMD is closely linked to the ability to prioritize tasks and allocate time efficiently (Cyril, 2015). Furthermore, students who recognize inefficiencies in their time use are more likely to adjust their habits, thereby developing stronger TMD (Cyril, 2015). These findings suggest that fostering BTP can enhance TMD, which in turn supports improved learning outcomes among high school students. Importantly, overcoming temporal biases and cultivating a BTP (Boniwell and Zimbardo, 2004) are essential for optimizing psychological functioning and achieving greater control over one’s life.

### Moderating effect of resilience on the mediating effect of TMD

In line with Diotaiuti et al. (2021), this study demonstrates how positive psychological traits, such as resilience and BTP, can enhance academic performance. However, further exploration is needed to understand the underlying mechanisms. The moderated mediation analysis revealed that resilience significantly moderated the mediating effect of TMD in the relationship between BTP and academic achievement. Specifically, this moderating effect was observed in the initial pathway of the mediation model, indicating that the strength of the association between BTP and TMD was contingent upon individuals’ resilience levels. More precisely, higher resilience amplified the positive influence of BTP on TMD, thereby strengthening the indirect effect of BTP on academic performance through improved TMD.

The moderating effect of resilience on the second half of the pathway was not significant, meaning that the relationship between TMD and academic performance was not affected by the level of resilience. Students’ time management skills greatly influence their academic performance and the skill is one of the predictors of academic performance (Nasrullah and Khan, 2015). Most of the students have medium level of time management skills and only very few have high level of time management skills (Yilmaz et al., 2006). The difference is not significant due to the generally low time management skills of high school students. This pattern was evident for all students, increasing resilience enhances the impact of BTP on time management disposition.

These findings emphasizing the importance of fostering positive psychological traits to enhance academic performance (Diotaiuti et al., 2021). The results suggest that the best outcomes would be produced with training in time-related skills while at the same time teaching or promoting resilience. The present study used the moderated mediation analyses to explain why and when resilience relates to BTP and time management disposition. These findings deepen our understanding of BTP translates their detrimental effects.

The GROW model states that resilience consists of four key components, “good emotions (G), reason and purpose (R), others and connection to the world (O), and wellness flexibility (W)” (Peccoralo et al., 2020, p. 192). Resilience is a process to harness resources to sustain well-being (Panter-Brick and Leckman, 2013) and perceive less psychological distress (Almeida, 2005; Friborg et al., 2006). Increased resilience can enhance mental health and promote individual development.

Resilience is common (Southwick et al., 2014) and a dynamic process (Luthar et al., 2000; Yates et al., 2003) that can be improved through training (Peccoralo et al., 2020). The level of resilience is the result of a balance of protective and risk factors, enhancing protective factors has been shown to be more effective than reducing risk factors to improve resilience (Lee et al., 2013; Zolkoski and Bullock, 2012). Furthermore, environmental factors play important roles in shaping personal resiliency (Roberts and Masten, 2004). One of the most important ways to foster resilience is to promote healthy family and community environments (Southwick et al., 2014). Creating a positive school climate can also promote positive relationships and social skills (Cohen, 2013) and increase levels of resilience.

### Limitations and suggestions

The main limitations of this study include the use of self-reported measures and the cross-sectional design, which limits causal inference. Future studies could adopt longitudinal designs and integrate objective data to strengthen the validity of the results.

For high school students, study time is limited. The problem associated with time constraints is learning how to balance time (Boniwell, 2005). As the best attitude toward time, BTP is closely related to a positive attitude toward life. When working toward a more balanced perception of time, it is possible to improve one’s functioning (Sobol and Jankowski, 2016), unlock one’s well-being (Boniwell et al., 2010), and enter into a state of enjoying being productive and creative (Boniwell and Zimbardo, 2004).

## Conclusion

This study provides empirical evidence on the importance of fostering BTP and resilience to improve academic performance. Academic performance represents merely one dimension of adolescent development. Future studies could explore educational interventions based on these psychological traits, as suggested by Diotaiuti et al. (2021), to assess their long-term impact.

## Data Availability

The raw data supporting the conclusions of this article will be made available by the authors, without undue reservation.
